# Insight into the First Draft Genome Sequence of the Genus *Sediminibacillus*, *Sediminibacillus halophilus* Strain NSP9.3

**DOI:** 10.1128/genomeA.01133-13

**Published:** 2014-01-09

**Authors:** Rinku Dey, Kamal Krishna Pal, Dharmesh Sherathia, Bhoomika Sukhadiya, Trupti Dalsania, Ilaxi Patel, Kinjal Savsani, Manesh Thomas, Sejal Vanpariya, Mona Mandaliya, Rupal Rupapara, Priya Rawal, Sucheta Ghorai, Sharmila Bhayani, Abhi Shah, Anil Kumar Saxena

**Affiliations:** Microbiology Section, Directorate of Groundnut Research, Junagadh, Gujarat, Indiaa; Division of Microbiology, Indian Agricultural Research Institute, New Delhi, Indiab

## Abstract

We report the 3.98-Mbp first draft genome sequence of *Sediminibacillus halophilus* strain NSP9.3, a moderate halophile isolated from a seasonal salt marsh of the Great Rann of Kutch, India. Exploring the genome of this organism will facilitate the understanding of the mechanism(s) of osmotolerance and survival in differential osmolarity.

## GENOME ANNOUNCEMENT

In the recent past, genomes of a number of halophiles, thriving in the saline environments of the Rann of Kutch, India, were sequenced to understand the mechanism(s) of osmotolerance (1–4). *Sediminibacillus halophilus* strain NSP9.3 (16S rRNA, GenBank accession no. JX518263), a moderately halophilic bacterium, was isolated from a seasonal salt marsh of the Great Rann of Kutch, India. This bacterium grows optimally at a 5% NaCl (range, 0 to 20%) concentration in growth medium at 37°C and 7.5 pH. The genome of *Sediminibacillus halophilus* strain NSP9.3 was sequenced to gain insight into its osmotolerance mechanism(s).

The whole genome of *Sediminibacillus halophilus* strain NSP9.3 (G+C content, 44.05%) was sequenced at Macrogen, Inc., South Korea, through Sequencher Tech Pvt., Ltd., Ahmedabad, India, using a Roche 454 genome sequencer (GS FLX), by both shotgun and 3-kb mate-paired library sequencing. Sequencing of shotgun and mate-paired libraries generated 782,771, 137,390, and 122,992 reads of 432,228,177, 60,630,498, and 53,887,735 bases with average read lengths of 552, 441, and 438 bp, respectively.

*De novo* assembly of reads using GS De Novo Assembler v 2.6 (5) gave approximately 134-fold coverage, with 10 scaffolds of 3,985,996 bp and 25 contigs of 3,983,638 bp. *N*_50_ scaffold lengths of 693,230 bp (smallest, 2,877 bp, and largest, 1,699,278 bp) and *N*_*50*_ contig lengths of 276,706 bp (smallest, 2,877 bp, and largest, 572,364 bp) were obtained. All assembly data were deposited in the DDBJ/EMBL/GenBank nucleotide sequence database.

The genome was annotated by using the RAST server (6), Glimmer 3 (7, 8), GeneMark (9, 10), the KEGG database (11), tRNAScan-SE (12), RNAmmer (13), and Signal P4.1 (14).

Using the different software tools, we predicted 4,232 coding sequences (CDS), 83 RNA-encoding genes (72 tRNAs and 11 rRNAs), 436 subsystems, and 60 signal peptides. Among the CDS, 2,445 are not in the subsystem (1,010 nonhypothetical, 1,435 hypothetical), whereas 1,787 CDS (1,679 nonhypothetical, 108 hypothetical) are in the subsystem. RAST annotation found involvement of 119 genes in stress responses. These genes included 30 in osmotic stress (2 in osmoregulation, 4 in ectoine biosynthesis and regulation, 23 in choline and betaine uptake and betaine biosynthesis, and 1 in synthesis of osmoregulatory periplasmic glucans), 40 in oxidative stress (9 in protection from reaction oxygen species [ROS], 20 in oxidative stress, 2 in the glutathione: redox cycle, 5 in redox-dependent regulation of nuclear processes, 2 in glutathione: nonredox reactions, and 2 in glutaredoxin reactions), 4 in cold shock, 19 in heat shock, and 26 in no subcategory. In addition, 12 genes in potassium homeostasis, 9 in glycerol and glycerol-3-phosphate uptake and utilization, 8 in mannitol utilization, 307 in amino acids and derivatives, 479 in carbohydrates utilization, etc. were identified. Moreover, a number of genes associated with different ABC transporters (map02010), like glycine betaine/proline (ProX, ProW, and ProV), osmoprotectant (OpuBC and OpuBA), phosphate (PstA, PstB, PstC, and PstS), branched-chain amino acids (LivF, LivG, LivH, LivK, and LivM), etc. and those in two-component systems (map02020) like salt stress-degradative enzymes (DegS and DegU), etc., have been mapped to KEGG pathways.

We are further exploring the first draft genome of *Sediminibacillus halophilus* NSP9.3 to understand its survival and osmoadaptation strategies.

### Nucleotide sequence accession numbers.

This whole-genome shotgun project has been deposited at DDBJ/EMBL/GenBank under the accession number AWXX00000000. The version described in this paper is version AWXX01000000.
